# Soil Microbial Diversity and Network Organization Respond to Land Use and Agricultural Inputs Worldwide

**DOI:** 10.1111/gcb.70984

**Published:** 2026-07-04

**Authors:** Zaki Saati‐Santamaría, Sergio Pérez‐Gorjón, Daniel Abel‐Schaad, Alberto Acedo‐Bécares, Francisca Alba‐Sánchez, Amanda Lucia Alves, Weronika Babinska‐Wensierska, Jorge Ariel Marfetán, Carolina Barroetaveña, Kara Barry, Francisco Beitia, Katerina Biniari, Elí Misael Bobadilla‐Peñaló, Gregory Bonito, Mihalis Boutaris, Victoria Bueno‐González, Guillermo Cabezas, Parharidis Charalambos, Ovidiu Copoț, Andrés de Errasti, Luis de Pedro Noriega, Philippe Delavault, Fernando Dianez‐Martínez, David Diez‐Méndez, Enrico Ercole, Abel Fernández‐Ruiz, Martina Ferraguti, Ana Laura Gallo, Paula García‐Fraile, Mario Garrido, David González del Pozo, Alina G. Greslebin, Gabriel Grilli, Edmundo Danilo Guilcapi‐Pacheco, Danny Haelewaters, Terry W. Henkel, Andrés Hirigoyen, Kentaro Hosaka, Pablo Yair Huais, Marja Jalli, Alfredo Justo, Marjo Keskitalo, Tommaso La Mantia, Aneta Lambevska, Ewald Langer, Corina Leconte, Ewa Lojkowska, David Marcos‐Vidal, Antonio J. Mendoza‐Fernández, Isabel Miralles‐Mellado, Lucía Molina, Norman Muzhinji, Minh N. Nguyen, Diego Nieto‐Lugilde, Alberto Nieto‐Palenzuela, André‐Ledoux Njouonkou, Sarah Norvell, Francisco J. Oficialdegui, Raúl Ortega Pérez, Ansa Palojärvi, Marcos Paradelo‐Pérez, Zunilda Pavone, Julio Peñas de Giles, Anna Maria Persiani, María Belén Pildain, Daniel Pinto‐Carrasco, Lucie Poulin, Jean‐Bernard Pouvreau, Paola Quatrini, David Rodríguez de la Cruz, Gonzalo M. Romano, Natalia Rosas‐Ramos, Francisca Ruano, Isabel Salcedo‐Larralde, Esteban Salmerón‐Sánchez, Katerina Sam, Cathy Sharp, Patricia Vieira Tiago, Ricardo Valenzuela, Aída M. Vasco‐Palacios, Mylonas Vasilis, María Laura Vélez, Alfredo Vizzini, Sergey Volobuev, Alan R. Wood, Pirjo Yli‐Hemminki, Nourou S. Yorou, Ivan V. Zmitrovich, Javier Bobo‐Pinilla

**Affiliations:** ^1^ Unidad de Excelencia Producción, Agrícola y Medioambiente (AGRIENVIRONMENT) Universidad de Salamanca Salamanca Spain; ^2^ Institute for Agrobiotechnology Research (CIALE) Universidad de Salamanca Salamanca Spain; ^3^ Departamento de Microbiología y Genética Universidad de Salamanca Salamanca Spain; ^4^ Laboratory of Fungal Genetics and Metabolism Institute of Microbiology of the Czech Academy of Sciences Prague Czech Republic; ^5^ Departamento de Botánica y Fisiología Vegetal Universidad de Salamanca Salamanca Spain; ^6^ Biobanco de ADN Vegetal Universidad de Salamanca Salamanca Spain; ^7^ Departamento de Botánica Universidad de Granada Granada Spain; ^8^ Biome Makers Inc Davis California USA; ^9^ Centro de Biociências (CB), Departamento de Micologia Universidade Federal de Pernambuco (UFPE) Recife Pernambuco Brazil; ^10^ Laboratory of Plant Protection and Biotechnology, Intercollegiate Faculty of Biotechnology University of Gdańsk and Medical University of Gdańsk Gdańsk Poland; ^11^ Centro de Investigación Forestal Andino‐Patagónico (CIEFAP) Esquel Chubut Argentina; ^12^ Tasmanian Institute of Agriculture University of Tasmania Hobart Tasmania Australia; ^13^ Instituto Valenciano de Investigaciones Agrarias (IVIA) Valencia Spain; ^14^ Department of Crop Science, Laboratory of Viticulture Agricultural University of Athens Athens Greece; ^15^ Herbario Erik Leonard Ekman (ELE) Universidad ISA Santiago Dominican Republic; ^16^ Department of Plant, Soil and Microbial Sciences Michigan State University East Lansing Michigan USA; ^17^ Kir‐Yianni Estate Naoussa Greece; ^18^ TRAGSA Madrid Spain; ^19^ Institute of Ecology and Earth Sciences University of Tartu Tartu Estonia; ^20^ Center for Macroecology, Evolution and Climate, Globe Institute University of Copenhagen, Universitetsparken 15D Copenhagen Denmark; ^21^ Facultad de Educación y Trabajo Social Universidad de Valladolid Valladolid Spain; ^22^ Unit in Biological Sciences and Biotechnologies, UMR 6286 Nantes Université, CNRS Nantes France; ^23^ Departamento de Agronomía y Centro de Investigación en Agrosistemas Intensivos Mediterráneos y Biotecnología Agroalimentaria (CIAIMBITAL) Universidad de Almería Almería Spain; ^24^ Department of Animal Ecology Netherlands Institute of Ecology (NIOO‐KNAW) Wageningen PB the Netherlands; ^25^ Biology Centre of the Czech Academy of Sciences, Institute of Entomology České Budějovice Czech Republic; ^26^ Faculty of Science University of South Bohemia České Budějovice Czech Republic; ^27^ Dipartimento di Scienze Della Vita e Biologia Dei Sistemi Università di Torino Torino Italy; ^28^ Department of Conservation Biology and Global Change Doñana Biological Station (CSIC) Seville Spain; ^29^ Consorcio de Investigación Biomédica en Red de Epidemiología y Salud Pública (CIBERESP) Madrid Spain; ^30^ Área de Biodiversidad y Conservación, Instituto de Investigación en Cambio Global (IICG‐URJC) Universidad Rey Juan Carlos Madrid Spain; ^31^ Eurofins MITOX Amsterdam the Netherlands; ^32^ Laboratorio de Bioprospección e Investigación Aplicada en Plantas y Hongos (LABIAPH), Facultad de Ciencias Naturales y Ciencias de la Salud Universidad Nacional de la Patagonia SJB Esquel Chubut Argentina; ^33^ Laboratorio de Micología Instituto Multidisciplinario de Biología Vegetal (UNC‐CONICET) Córdoba Argentina; ^34^ Escuela Superior Politécnica de Chimborazo (ESPOCH) Riobamba Ecuador; ^35^ Faculty of Sciences, Department of Biology, Research Group Mycology Ghent University Ghent Belgium; ^36^ Department of Biological Sciences Humboldt State University California USA; ^37^ Instituto Nacional de Investigación Agropecuaria (INIA), Camino del Colorado Canelones Uruguay; ^38^ Department of Botany National Museum of Nature and Science Ibaraki Japan; ^39^ Facultad de Ciencias Exactas, Físicas y Naturales Universidad Nacional de Córdoba Córdoba Argentina; ^40^ Natural Resources Institute Finland (Luke) Helsinki Finland; ^41^ New Brunswick Museum Saint John New Brunswick Canada; ^42^ Dipartimento SAAF ‐ Scienze Agrarie, Alimentari e Forestali Università Degli Studi di Palermo Palermo Italy; ^43^ Department of Plant and Fungal Diversity and Resources Bulgarian Academy of Sciences, Institute of Biodiversity and Ecosystem Research Sofia Bulgaria; ^44^ Department of Ecology University Kassel Kassel Germany; ^45^ Establecimiento Las Marías Corrientes Argentina; ^46^ Department of Plant Sciences University of the Free State Bloemfontein South Africa; ^47^ Faculty of Environmental Sciences University of Science, Vietnam National University Hanoi Vietnam; ^48^ Departamento de Botánica, Ecología y Fisiología Vegetal, Campus de Excelencia Internacional Agroalimentario CeiA3 Universidad de Córdoba Córdoba Spain; ^49^ Bakcheia Mundo Enologico C.B Valladolid Spain; ^50^ Department of Plant Sciences, Faculty of Science The University of Bamenda Bamenda Cameroon; ^51^ Faculty of Fisheries and Protection of Waters, South Bohemian Research Center of Aquaculture and Biodiversity of Hydrocenoses University of South Bohemia in Ceske Budejovice Vodňany Czech Republic; ^52^ Natural Resources Institute University of Greenwich Kent UK; ^53^ Manexa S.A Buenos Aires Argentina; ^54^ Dipartimento di Biologia Ambientale Sapienza Università di Roma Roma Italy; ^55^ Dipartimento di Scienze Della Terra e del Mare (DISTEM) Università Degli Studi di Palermo Palermo Italy; ^56^ MycoThink, ABIOINNOVA Incubadora de Empresas de Alta Tecnología en el Sector biotecnológico Salamanca Spain; ^57^ Departamento de Biología Animal, Ecología, Parasitología, Edafología y Química Agrícola Universidad de Salamanca Salamanca Spain; ^58^ Departamento de Zoología Universidad de Granada Granada Spain; ^59^ Departamento de Biología Vegetal y Ecología Universidad del País Vaco (UPV/EHU) Bilbao Spain; ^60^ Departamento de Biología y Geología, ENGLOBA y CEIMAR Universidad de Almería Almería Spain; ^61^ Natural History Museum of Zimbabwe Bulawayo Zimbabwe; ^62^ Laboratorio de Micología, Departamento de Botánica, Escuela Nacional de Ciencias Biológicas Instituto Politécnico Nacional Ciudad de México México; ^63^ Grupo Biomicro, Escuela de Microbiología Universidad de Antioquia UdeA Medellín Colombia; ^64^ Komarov Botanical Institute, Saint Petersburg St Petersburg Russia; ^65^ College of Agriculture, Engineering and Science University of KwaZulu‐Natal Stellenbosch South Africa; ^66^ Tropical Mycology and Plant‐Soil Fungi Interactions, Faculty of Agronomy University of Parakou, Benin Parakou Benin; ^67^ Departamento de Didáctica de Las Ciencias Experimentales, Sociales y de la Matemática Universidad de Valladolid Valladolid Spain

**Keywords:** agricultural management, bacteria, fertilization, fungi, microbial diversity, microbiome, natural habitats, network topology, pesticides, soil microbial communities

## Abstract

Soil microbiomes are critical for ecosystem functioning, yet the global influences of climate and agricultural practices on their diversity and structure remain incompletely characterized. Here we analyzed 1921 soil samples from 33 countries worldwide across diverse biomes to assess how climate gradients and agricultural inputs, including pesticides and fertilizers, shape prokaryotic and fungal communities. We found that microbial diversity peaks at intermediate temperatures and differs markedly between natural and agricultural soils, with agriculture increasing microbial diversity while altering community composition and ecological guilds. Pesticide use selectively reduced bacterial diversity and shifted fungal guilds, decreasing ectomycorrhizal fungi while increasing saprotrophs, whereas fertilization reduced microbial network cohesion, with organic and inorganic fertilizers eliciting distinct community responses. These findings reveal that climatic factors and agricultural management jointly influence soil microbial diversity, community structure, and network connectivity, with implications for soil health and ecosystem resilience in managed landscapes. Overall, our results demonstrate that agricultural practices, including the use of pesticides and both organic and inorganic fertilizers, act as strong ecological filters that reshape soil microbiomes worldwide—enhancing apparent diversity but driving a functional shift toward less mutualistic, more fragmented, and potentially less resilient communities.

## Graphical abstract

Based on a global analysis of 1921 soil samples, we show that agricultural management acts as a powerful ecological filter reshaping microbial communities. Land use and inputs—pesticides and fertilizers—drive a functional shift toward opportunistic pathogens and fragmented, less connected microbial networks, potentially compromising soil ecosystem resilience and stability on a global scale.

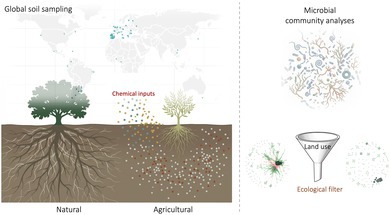

## 1 Introduction

Soil microbiomes play a central role in ecosystem functioning, influencing nutrient cycling, plant health, and ecosystem productivity (Hartmann and Six 2023; Philippot et al. 2024; Muhammad et al. 2025; Singh et al. 2025). Our understanding of soil microbial diversity has progressed rapidly over the last decade, offering new insights into how microbial communities respond to climate change, land use, and management practices (Peng et al. 2024; Peddle et al. 2024; Khashi u Rahman et al. 2025; Rodríguez del Río et al. 2025). It is now well established that soil microbial communities respond to external drivers both functionally and compositionally, with bacteria and fungi often exhibiting divergent patterns (Bahram et al. 2018; De Vries et al. 2018; Whitman et al. 2019; Baldrian et al. 2023; Sasse et al. 2018; Habtewold et al. 2020; Saati‐Santamaría et al. 2023) Furthermore, land management, especially the contrast between natural and agricultural systems, strongly influences soil microbial diversity and composition. Managed soils often show higher microbial richness but host distinct communities shaped by disturbance and heterogeneity (Romdhane et al. 2022; Labouyrie et al. 2023).

Despite this progress, most large‐scale studies to date have focused primarily on environmental gradients or soil physicochemical parameters in natural systems, often overlooking key anthropogenic factors such as pesticide or fertilizer inputs (Crowther et al. 2019; Větrovský et al. 2019; Knight et al. 2024). Moreover, existing surveys are frequently limited in geographic scope or concentrate on a narrow range of common crops, underrepresenting the diversity of agroecosystems worldwide. Together, this has led to a fragmented understanding of how agriculture shapes microbial diversity across different soil types, climates, and crop species. For example, a recent study across 60 agricultural soils in Switzerland found that specific pesticide residues, including glyphosate, carbendazim, and 2‐hydroxyatrazine, were associated with significant shifts in bacterial and fungal diversity, and influenced the abundance of microbial taxa involved in nitrogen cycling (Walder et al. 2022). Experimental evidence from 22 soils in Italy indicates that pesticide applications can differentially affect key soil enzymatic activities, such as those involved in carbon, nitrogen, and phosphorus cycling, with some compounds stimulating enzyme activity in some soils and others exerting inhibitory effects (Sannino and Gianfreda 2001). Regarding fertilizer inputs, it was found that both mineral and organomineral fertilizers can shape microbial community composition, sometimes reducing overall richness, while enriching bacterial taxa involved in nitrogen cycling and organic matter turnover (e.g., *Azospirillum*, *Phenylobacterium*) (Oliveira et al. 2025).

Long‐term field experiments further reveal that manure and chemical fertilization can differentially affect soil antibiotic resistance gene pools depending on soil type and redox conditions (Wang et al. 2018), while nutrient additions in a boreal pine forest modulate fungal communities and decomposition rates in ways that ultimately constrain or enhance soil carbon sequestration (Richy et al. 2024). Long‐term trials in Czechia show that fertilization regimes significantly alter both bacterial and fungal communities, with fungi showing stronger sensitivity to treatment type (Kracmarova et al. 2022). Mineral fertilizer application tends to increase both microbial biomass and soil organic carbon in paddy rice systems (Geisseler et al. 2017). These changes were suggested to be mediated by enhanced crop productivity and carbon inputs, rather than by consistent shifts in specific microbial groups (Geisseler et al. 2017). Global meta‐analyzes have shown that organic fertilization can simultaneously enhance plant biomass, soil organic carbon, and plant diversity—particularly in grasslands—positioning it as a promising nature‐based solution to support multiple ecosystem services (Shi et al. 2024); however, these benefits have rarely been directly linked to shifts in soil microbial communities. Also, long‐term application of organic fertilizer in tea orchards reduces heavy metal accumulation in soil and leaves, improves soil pH and tea quality, and selectively alters rhizosphere bacterial communities by enriching beneficial taxa (Lin et al. 2019). To date, a global, cross‐system assessment integrating multiple plant hosts, climatic zones, and management regimes is still missing.

Here, we present results of a global environmental microbiome study which sought to address the following questions: (I) How do soil fungal and bacterial communities differ between natural and agricultural ecosystems across climatic and plant‐host contexts? (II) To what extent do pesticides and fertilizers alter the taxonomic and functional structure of soil microbiomes? (III) How do different agricultural inputs affect microbial network organization and the abundance of key ecological guilds? To answer these questions, we present a global analysis of soil microbiomes across 1921 samples from both natural and agricultural soils, spanning a broad range of plant‐associated habitats. These include soils from ecosystems dominated by *Quercus ilex* , *Ilex paraguariensis* , and pastures, as well as cultivated lands supporting *Vitis vinifera* , *Prunus dulcis* , *Prunus avium*, *Olea europaea* , *Pistacia vera* , *Actinidia deliciosa* , and other economically and ecologically relevant species. Notably, several of the sampled host‐taxa have been rarely included in global‐scale microbiome research. We systematically compare microbial diversity, taxonomic composition, and co‐occurrence network structure between natural and agricultural soils. Within agricultural systems, we further dissect the impact of pesticide application, fertilization status, and fertilizer type (inorganic vs. organic) on microbial community structure. By integrating ecological and management factors across diverse biomes and plant hosts, our study provides a comprehensive view of how agricultural practices reshape the global soil microbiome.

## 2 Methods

### 2.1 Soil Sampling and Processing

This study analyzed a total of 1921 topsoil samples collected worldwide in 2021. Sampling was conducted by collaborators following standardized field protocols. Samples corresponding to bulk topsoil were collected from the upper layer in the first 10 to 15 cm after removing the most superficial layer of leaves and humus (approximately 0–10 cm). For each site, several subsamples (typically 5–20, depending on field size and land use) were obtained to capture local‐scale heterogeneity and combined in a sterile plastic bag in the field to obtain a representative composite sample capturing local‐scale heterogeneity. After thorough homogenization, coarse roots and stones were removed, and the soil was ground into a fine powder using bead beating. Composite samples were then transferred into sterile Falcon tubes (76 × 20 mm), maintained at 4°C in a portable cooler, and shipped for molecular analysis to the Biome Makers laboratory in Valladolid, Spain, or Sacramento, USA, depending on the proximity of the sampling sites.

Upon arrival, samples were stored at −20°C or −80°C until DNA extraction. DNA was extracted using a bead‐beating protocol with the DNeasy PowerLyzer PowerSoil Kit (Qiagen), and subsequently analyzed through the BeCrop platform (patent WO2017096385; Becares and Fernandez 2017). The use of this standardized, mechanical‐lysis approach ensured comparability across sites and maximized recovery of microbial diversity from the soil matrix. A detailed list of samples and their geographic origins is provided in Table S1.

Amplification of microbial marker genes targeted the 16S rRNA gene (V4 region) for prokaryotes and the ITS1 region for fungi, using BeCrop custom primer sets (patent WO2017096385; Becares and Fernandez 2017). All PCRs were prepared under UV‐sterilized conditions, and negative controls were included in each run. All libraries were prepared following the two‐step PCR Illumina protocol. Sequencing was carried out on an Illumina MiSeq system (Illumina, San Diego, CA, USA), generating 2 × 301 bp paired‐end reads.

### 2.2 Bioinformatic Processing and Diversity Analyzes

We analyzed amplicon sequencing data from the 16S rRNA gene and the ITS region using QIIME2 (2023.9 release) (Bolyen et al. 2019). Quality filtering and denoising were performed with the DADA2 plugin, which generated amplicon sequence variants (ASVs). For bacterial sequences, forward reads were trimmed and truncated at 17 and 240 nucleotide positions, respectively, and reverse ones at 21 and 100 nucleotides, in order to preserve the highest‐quality regions and ensure optimal merging of forward and reverse reads. ITS reads were trimmed and truncated at 16 and 206 nucleotide positions, respectively. Alpha and beta diversity metrics were calculated with QIIME2 plugins, enabling statistical comparisons between sample categories. Shannon index (H′) was calculated using QIIME2 based on the following equation:

$$H^{\prime }=-\sum_{\left\{i=1\right\}}^{\left\{S\right\}}p_{i}\backslash \ln\left(p_{i}\right)$$

where *S* is the total number of taxa in the community and *pᵢ* is the relative abundance of taxon *i*. This metric incorporates both richness and evenness of the community (Shannon 1948; Magurran 2004). Shannon entropy values were respectively compared using the Kruskal–Wallis test, while Bray–Curtis distances were analyzed with a permutational ANOVA (pseudo‐*F*, 999 permutations).

For stratified analyzes, samples were additionally grouped according to two independent metadata dimensions: (i) biome classification and (ii) land‐use/management category. Biome categories were assigned based on externally provided metadata and corresponded to broad ecological regions (e.g., boreal forest, grasslands, Mediterranean, shrublands, temperate coniferous forest). Land‐use/management categories reflected local site characteristics, including crop type and agricultural management practices (e.g., fertilization and pesticide application where available). These two classification schemes were used for distinct analytical purposes.

The contribution of management variables to microbial community structure was assessed using permutational multivariate analysis of variance (PERMANOVA), implemented through the adonis2 function from the vegan R package (v2.7–1) (Dixon 2003). We used Bray–Curtis dissimilarity matrices and a model including multiple predictors such as biome, host species, and input use (e.g., fertilizers, pesticides), with 999 permutations. To ensure that the order of predictors did not affect the results, we set the argument by = “margin”.

Principal coordinates analysis (PCoA) was performed using Bray–Curtis dissimilarities to visualize patterns of microbial community composition. The ordination was computed via classical multidimensional scaling (cmdscale n in the stats package of base R), retaining the two principal axes. Sample coordinates were merged with associated metadata for visualization. Points were plotted by biome category, with colors and transparency adjusted to enhance interpretability.

Bacterial taxonomy was assigned using a classifier trained on the SILVA database (release 132, reference Operational Taxonomic Units (OTUs) clustered at 99% sequence identity) (Quast et al. 2012), after extracting and filtering database sequences based on the specific 16S rRNA primers to retain target sequences of 250–350 bp. Fungal sequences were classified using the UNITE database (v9), and Amplicon Sequence Variants (ASVs) identified as chloroplast or mitochondrial in the 16S rRNA dataset were excluded from further analysis.

Fungal taxonomic assignments obtained from ITS amplicon sequencing were first aggregated at the genus level. Each genus was subsequently assigned to ecological guilds using the FungalTraits database (v1.2) (Põlme et al. 2020), a curated trait database compiled from extensive literature curation and expert knowledge on fungal ecology. Assignments were performed by exact taxonomic matching between our genus‐level taxonomy and the FungalTraits reference classifications. Taxa without a corresponding entry in FungalTraits were classified as “unassigned”. Relative abundances of ecological guilds were calculated by summing the relative abundances of all genera assigned to each functional category within each sample. These values were used to compare functional composition across land‐use types.

We assessed the enrichment or depletion of microbial genera across four comparisons: (i) natural vs. agricultural soils; and, within agricultural soils, (ii) pesticide‐treated versus untreated sites, (iii) fertilized versus unfertilized sites, and (iv) treated with inorganic versus organic fertilizers. Given the compositional nature of microbiome data, we applied the Analysis of Compositions of Microbiomes with Bias Correction (ANCOM‐BC) framework (Lin and Peddada 2020), which adjusts for sampling bias and allows for robust estimation of differential abundance. Instead of relying solely on *p*‐values, ANCOM‐BC provides bias‐corrected log‐fold changes and controls for false discovery rate (FDR).

### 2.3 Generalized Additive Models

To assess the relationship between alpha diversity (Shannon entropy) and climatic variables, we fitted generalized additive models (GAMs) for each climate variable obtained from the WorldClim dataset (v2) (Fick and Hijmans 2017). These analyzes were performed in R using the *mgcv* package (v1.9.3) (Wood 2017). Separate models were constructed for bacterial (16S) and fungal (ITS) communities using Shannon entropy as the response variable and each climatic variable as a smoothed predictor. We used cubic regression splines with a basis dimension of 10 (*k* = 10) to balance flexibility and avoid overfitting. The models were estimated with restricted maximum likelihood (REML) to optimize smoothness selection. Model performance was evaluated using the coefficient of determination (*R* ^2^), which quantifies the proportion of variance in Shannon entropy explained by each climatic variable. To visualize these relationships, scatter plots were generated with GAM‐derived smooth curves and confidence intervals, with data points color‐coded by marker (16S or ITS). Density‐enhanced visualizations for selected climate variables (e.g., BIO1, BIO9) were produced using *ggMarginal* from *ggExtra* (v0.10.1) to illustrate the joint distribution of diversity values and climatic predictors. Each climatic variable was presented as a separate facet to facilitate comparative interpretation.

### 2.4 Microbial Co‐Occurrence Networks

Microbial co‐occurrence networks were inferred using the package “NetCoMi” (v1.1.0) (Peschel et al. 2021) in R (v4.4.0). The input tables consisted of ASV counts filtered as follows: ASVs with total abundance < 100 were discarded, and only taxa present in at least 20% of the samples were retained. Network construction was performed using SparCC as the association measure. Sparsification was applied using a correlation threshold (sparsMethod = “threshold”, thresh = 0.3). In this study, “network organization” is considered as the overall topology of microbial co‐occurrence networks, summarized by global metrics such as network density, clustering coefficient, and modularity. Network topology was analyzed via netAnalyze using the fast‐greedy community detection algorithm (clustMethod = “cluster_fast_greedy”) and computing centrality measures for the largest connected component (centrLCC = TRUE). Networks were visualized using the spring layout, with node size proportional to degree and without hub annotation. To assess robustness and compare structural features between conditions, we performed a bootstrap‐based resampling procedure. For each condition, 50 network replicates were generated by randomly removing 5% of the ASVs in each iteration. For each replicate, three global metrics were extracted: modularity, average clustering coefficient, and network density.

### 2.5 Statistics and Graphical Representations

For all group (i.e., categorical variables) comparisons in this study, we first assessed normality and homogeneity of variances using the Shapiro–Wilk and Levene's tests, respectively. When both assumptions were satisfied, analysis of variance (ANOVA) was applied, followed by Tukey's HSD post hoc test to identify significant differences between groups. If at least one assumption was violated, non‐parametric tests were used according to the comparison type: for comparisons among more than two groups, the Kruskal–Wallis test was followed by Dunn's test for multiple comparisons; for comparisons between two groups, the Wilcoxon rank‐sum test was used, or the Brunner–Munzel test when heterogeneity of variances and non‐normality were present. All statistical analyzes were performed in R (v4.4.0) using standard packages including “stats,” “FSA,” and “lawstat.”

To visualize the geographic distribution of sampling locations, we generated a world map using the R package “mapdata” (v2.3.1) (Becker and Wilks 1995). The base map was obtained from the worldHires dataset (Becker and Wilks 1995). Sampling locations were plotted as points using the package “ggplot2”. In addition, “ggplot2” was used to generate boxplots, violin plots, and volcano plots (Wickham 2011).

## 3 Results

### 3.1 Climate Gradients Explain Non‐Linear and Modest Variation in Continental‐Scale Microbial Diversity ( *R* ^2^ = 0.09–0.21)

**FIGURE 1:**
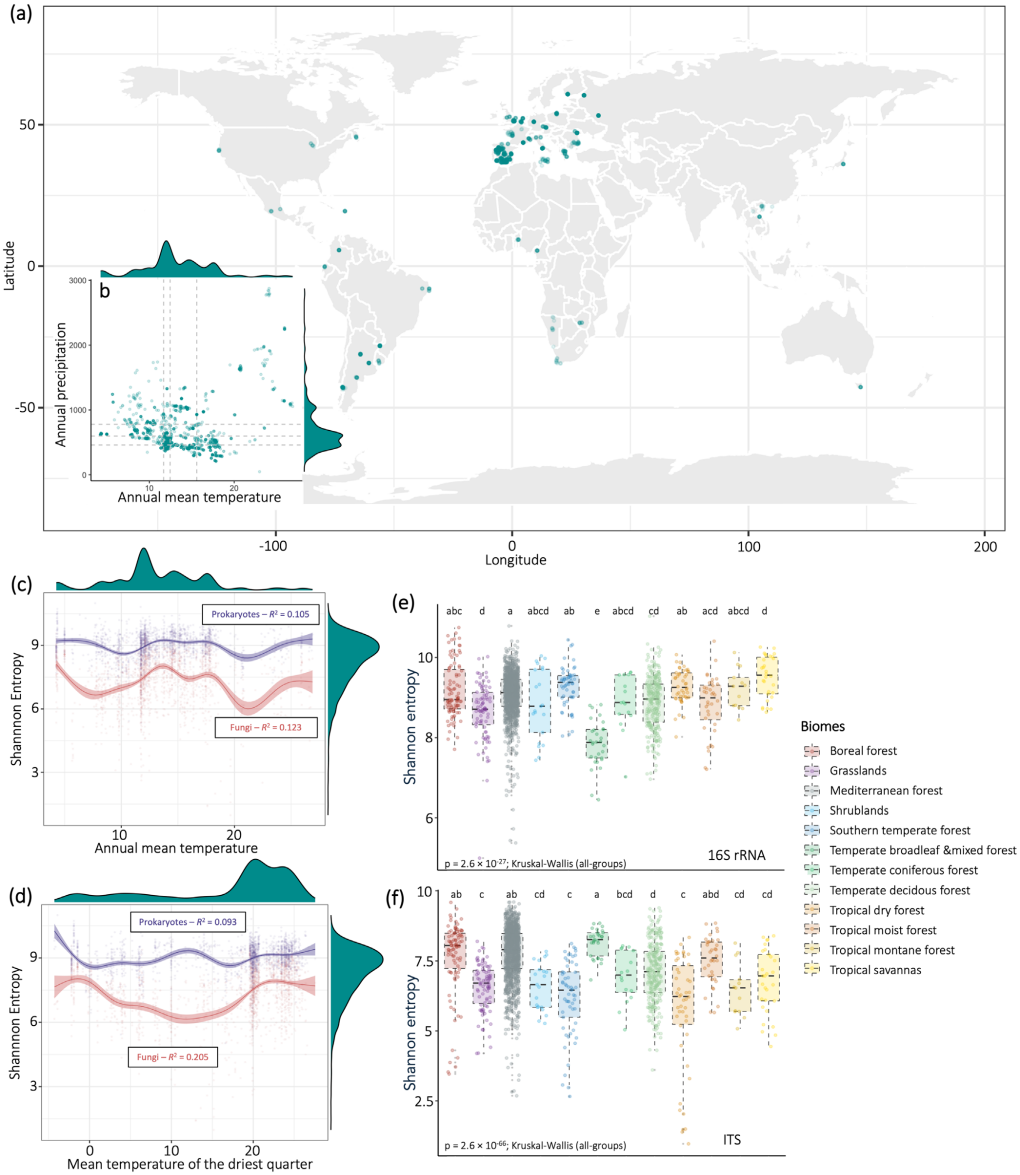
Global patterns of soil microbial diversity across sampling sites. (a) Geographic distribution of soil samples sequenced for bacterial/archaeal (16S rRNA gene) and fungal (ITS) communities, shown by latitude and longitude. Map lines delineate study areas and do not necessarily depict accepted national boundaries. (b) Climate space of the sampled sites, represented by annual mean temperature versus annual precipitation. (c) Relationships between Shannon entropy and annual mean temperature for 16S and ITS datasets, modeled using generalized additive models (GAMs). (d) Relationships between Shannon entropy and mean temperature of the driest quarter for 16S and ITS datasets (GAMs). Other bioclimatic variables and their associations with diversity are shown in Figure S1. (e, f) Boxplots of Shannon entropy across major terrestrial biomes for 16S (e) and ITS (f) datasets. Biome categories are based on ecoregion classifications provided in the sample metadata and do not include land‐use categories. Agricultural soils were analyzed separately as a land‐use category spanning multiple biomes and are therefore not shown in this panel.

We analyzed a global dataset of soil samples from 33 countries and diverse ecosystems and biomes (Figure 1a, Table S1), with 1915 samples sequenced for 16S rRNA gene and 1911 for ITS1 to profile prokaryotic and fungal communities, respectively (Tables S2 and S3). This extensive dataset spans broad gradients of temperature, precipitation, and aridity, enabling a comprehensive assessment of how microbial diversity and community composition respond to major climatic drivers. However, the distribution of samples was not uniform across the globe. Most sites fell within temperate and Mediterranean climates—characterized by mean annual temperatures between 10°C–20°C and annual precipitation below 1000 mm (Figure 1b, Table S1)—reflecting the fact that agricultural activity, and thus sampling effort, was concentrated in these regions, while a smaller subset of samples extends into colder or drier areas.

We analyzed how microbial α‐diversity varies along these temperature and precipitation gradients. Based on univariate GAMs across all bioclimatic predictors (Table S4, Figure S1), BIO9 (mean temperature of the driest quarter; *R* ^2^ = 0.094 in 16S; 0.206 in ITS) emerged as the strongest predictor for fungal diversity (Table S4, Figure 1d). Along this gradient, divergent trends emerged between prokaryotes and fungi: prokaryotic diversity again peaked at intermediate values (~10°C), whereas fungal diversity showed a clear minimum at similar temperatures. This contrasting behavior may reflect domain‐specific strategies, with bacterial/archaeal communities potentially benefiting from moderate warming during dry seasons, while fungi may be more constrained by seasonal drought conditions. BIO11 (mean temperature of the coldest quarter; *R* ^2^ = 0.115 in 16S; 0.111 in ITS) showed the highest explanatory power for prokaryotic diversity (Table S4, Figure S1). Isothermality (BIO3) consistently ranked as the second most important predictor in both datasets (*R* ^2^ = 0.109 in 16S; 0.191 in ITS). Both prokaryotic and fungal Shannon entropy also exhibited broadly similar non‐linear responses to mean annual temperature (BIO1; *R* ^2^ = 0.105 in 16S; 0.123 in ITS) (Figure 1c), peaking at intermediate temperatures (~10°C–15°C), declining at moderately colder and warmer conditions, and showing an apparent increase toward the extremes of the gradient, although the low number of observations at these extremes prevents robust inference. Generalized additive models (GAMs) confirmed these complex, non‐unimodal trends (Figure 1c). We interpret this pattern as likely arising from ecological filtering at suboptimal temperatures combined with niche specialization or long‐term adaptation driving increased diversity at climatic extremes. BIO1 (Annual Mean Temperature), although not among the strongest individual predictors, was retained as a general descriptor of mean climatic conditions and showed consistent non‐linear responses across both prokaryotic and fungal communities. Across the remaining bioclimatic variables, patterns of 16S rRNA and ITS diversity were generally concordant (Figure S1).

In addition, significant differences in Shannon entropy were observed across biomes (Figure 1e). While most biomes followed parallel trends for 16S and ITS datasets, some striking exceptions were observed. Notably, temperate broadleaf and mixed forests displayed the lowest bacterial diversity but the highest fungal diversity among all biomes, highlighting domain‐specific ecological constraints. Conversely, in most other biomes, bacterial and fungal diversity followed similar trends, indicating that large‐scale biogeographic factors can impact microbial groups in both shared and distinct ways.

### 3.2 Contrasting Microbial Communities and Functional Traits Between Natural and Agricultural Soils

**FIGURE 2:**
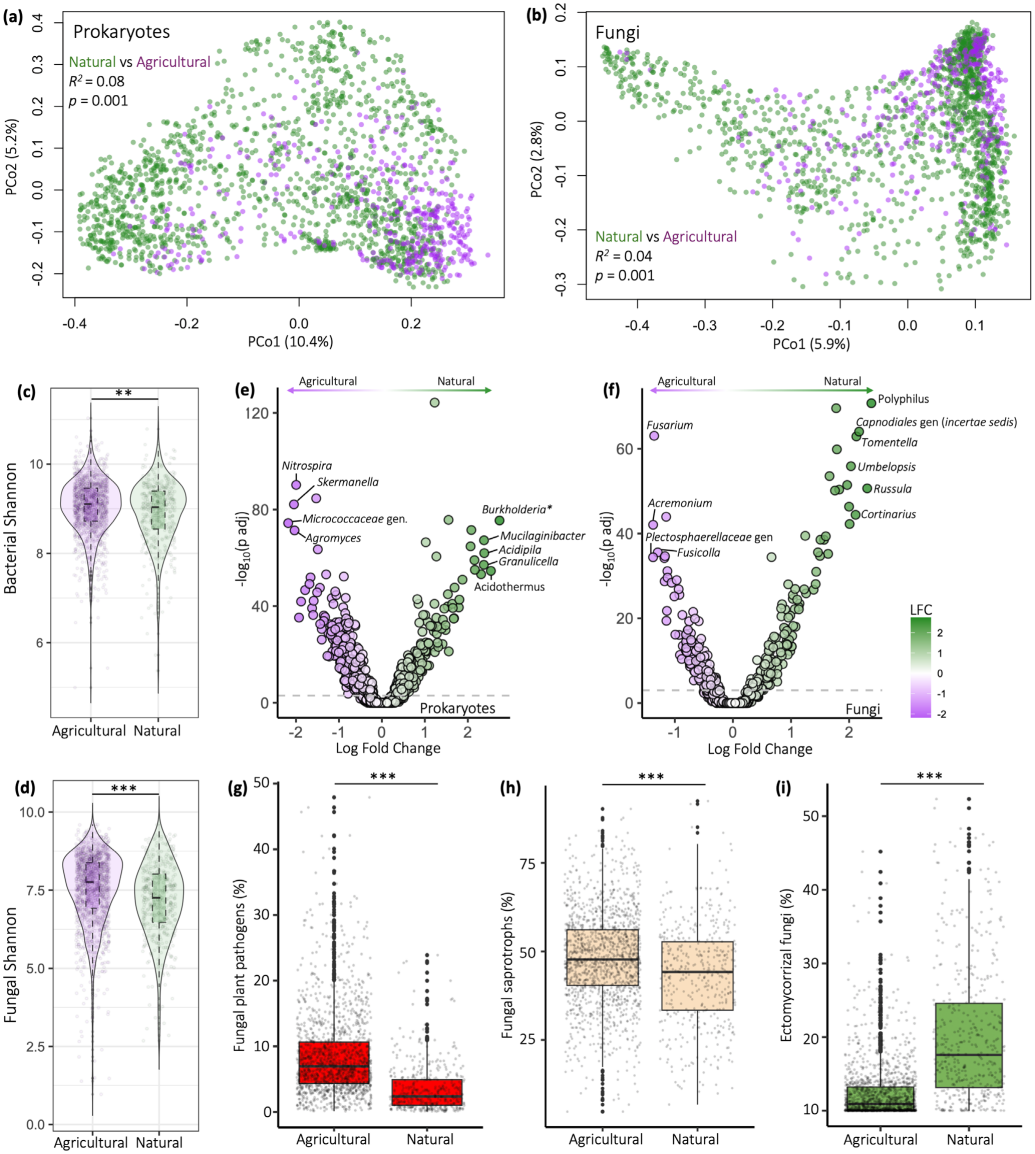
Effects of land use on global soil microbial diversity and composition. (a–b) Principal coordinates analysis (PCoA) of bacterial/archaeal (16S) (a) and fungal (ITS) (b) communities, colored by natural versus agricultural soils. (c–d) Boxplots of Shannon entropy for bacteria/archaea (c) and fungi (d) in natural and agricultural soils. (e–f) Volcano plots showing genera significantly enriched in natural or agricultural soils based on ANCOM‐BC analysis for 16S rRNA gene (e) and ITS (f). Asterisks (*) indicate undescribed genera assigned only to higher taxonomic ranks. Grey dashed lines represent the adjusted significance threshold (*p*‐adj < 0.001). (g–i) Boxplots of the relative abundance of fungal functional traits: (g) plant pathogens, (h) saprotrophs, and (i) ectomycorrhizal fungi, comparing natural and agricultural soils.

Principal coordinates analyzes (PCoA) based on Bray–Curtis dissimilarities revealed clear compositional differences between land‐use types for both prokaryotic (16S) and fungal (ITS) communities (Figure 2a,b), with PERMANOVA indicating significant separation (adonis *R* ^2^ = 0.08 and *R* ^2^ = 0.04 for prokaryotes and fungi respectively, *p* < 0.001 for both datasets), although these factors explain a modest proportion of the total variance. Alpha diversity was also significantly different between groups: agricultural soils (*n* = 1482) harbored higher Shannon entropy than natural soils (*n* = 439) for both 16S (*p* = 0.002, Brunner–Munzel test) and ITS datasets (*p* = 1.0 × 10^−10^, Wilcoxon test) (Figure 2c,d).

ANCOM‐BC analysis between land‐use types identified numerous bacterial and fungal genera driving these compositional shifts (adjusted *p* < 0.001; Figure 2e,f). Bacterial genera enriched in natural soils included *Burkholderia*‐complex, *Mucilaginibacter*, *Granulicella*, *Acidothermus*, and *Acidipila*, while among fungi, we detected *Polyphilus*, unclassified *Capnodiales* (incertae sedis), *Tomentella*, *Umbelopsis, Russula*, and *Cortinarius*. In contrast, agricultural soils were enriched in copiotrophic or plant‐associated fungal genera such as *Fusarium*, *Acremonium*, *Fusicolla* (Figure 2f), and bacteria from genera *Nitrospira*, *Skermanella*, *Agromyces*, and unclassified *Micrococcaceae* (Figure 2e). Although no fungal genera associated with agricultural soils exceeded a large log fold change, several showed consistent and statistically robust enrichment.

Regarding the distribution of ecological guilds among fungal communities (Figure 2g–i), agricultural soils harbored significantly higher relative abundances of plant pathogens (*p* < 0.001) and saprotrophs (*p* < 0.001), whereas ectomycorrhizal fungi were more abundant in natural soils (*p* < 0.001), highlighting differences in the distribution of ecological guilds. between land‐use types. This pattern may also reflect, at least in part, a sampling bias, since many cultivated plants are not typically associated with ectomycorrhizal symbioses.

### 3.3 Pesticide Application Alters Microbial Diversity, Ecological Guilds, and Community Structure in Agricultural Soils

**FIGURE 3:**
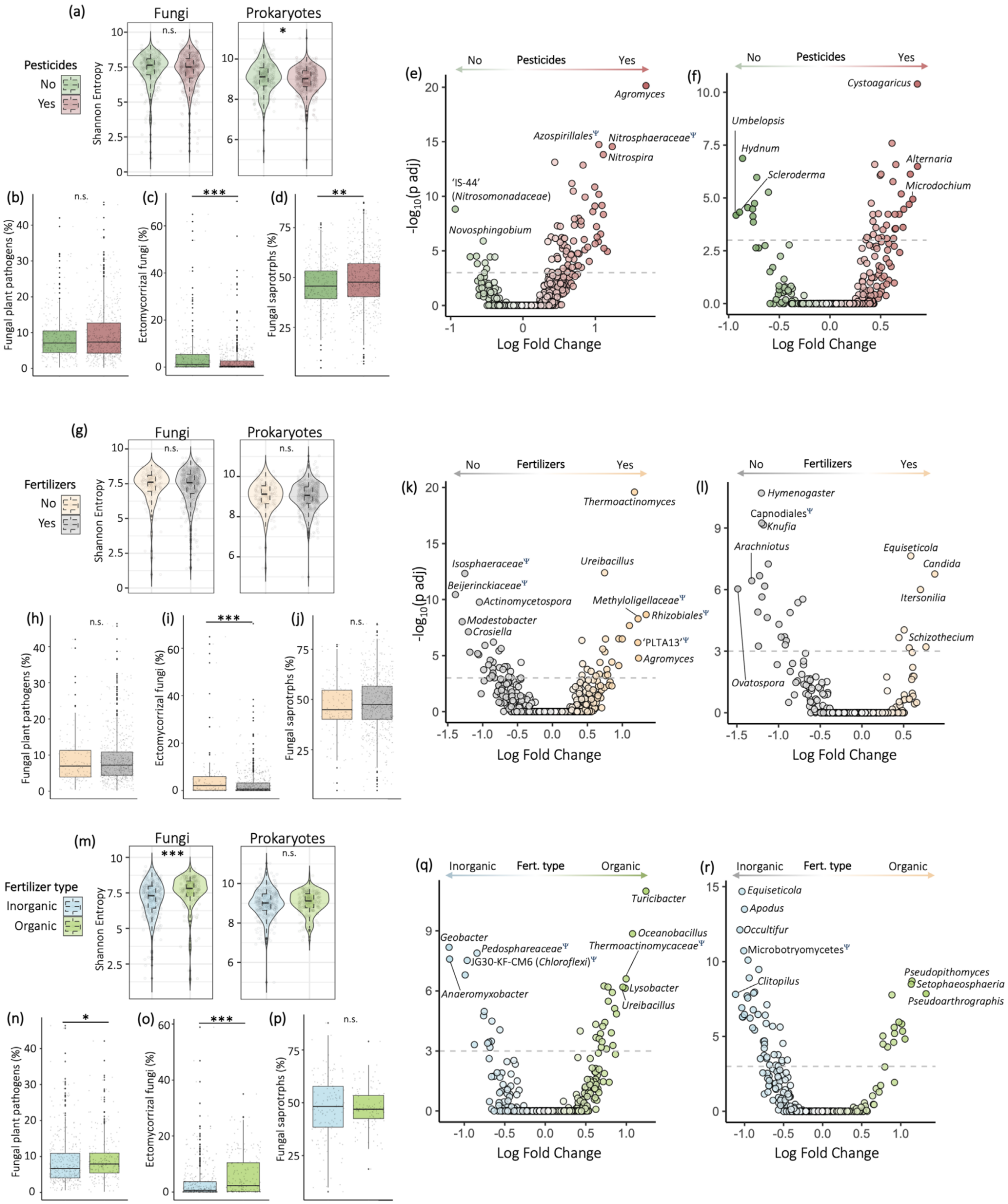
Effects of agricultural practices on soil microbial diversity, functional traits, and taxonomic composition. (a) Shannon entropy of bacterial/archaeal (16S rRNA gene) and fungal (ITS) communities in agricultural soils with and without pesticide use. (b–d) Relative abundance of fungal functional traits—(b) plant pathogens, (c) ectomycorrhizal fungi, and (d) saprotrophs—in soils with versus without pesticide use. (e–f) Volcano plots showing genera significantly enriched in soils with vs. without pesticide use based on ANCOM‐BC analysis of 16S rRNA gene (e) and ITS (f) datasets. (g–j) Same comparisons as in panels a–d but for agricultural soils with vs. without fertilization. (k–l) Volcano plots of genera enriched in fertilized versus unfertilized soils for 16S rRNA gene (*k*) and ITS (l). (m–p) Same comparisons as in panels a–d but for soils treated with inorganic versus organic fertilizers. (q–r) Volcano plots of genera enriched in inorganic versus organic fertilizer treatments for 16S rRNA gene (q) and ITS (r). Asterisks (*) denote undescribed genera assigned only to higher taxonomic ranks. Grey dashed lines represent the adjusted significance threshold (*p*‐adj < 0.001) in all volcano plots. For boxplots in panels A–R: *p < 0.05; **p < 0.01; ***p < 0.001; n.s., not significant.

We evaluated the impact of pesticide application on microbial communities within agricultural soils. Although fungal diversity (Shannon entropy) remained unaffected (Figure 3a), prokaryotic diversity significantly decreased in pesticide‐treated agricultural soils (*p* = 0.019; pesticide use *n* = 536, no use *n* = 618), indicating a selective effect on bacterial community structure. PERMANOVA analyzes revealed that pesticide use explained only a very small fraction of the overall community variation (*R* ^2^ = 0.009 for 16S rRNA and 0.005 for ITS, both *p* < 0.001), suggesting that pesticide application was only weakly associated with overall microbiome compositional shifts across sites.

At the functional level, no differences were observed in the relative abundance of fungal plant pathogens between treatments (Figure 3b). However, significant changes were detected in specific fungal ecological guilds: ectomycorrhizal fungi were significantly reduced in pesticide‐treated soils (*p* = 5.7 × 10^−5^; Figure 3c), while saprotrophic fungi increased (*p* = 0.008; Figure 3d). These changes indicate a redistribution of fungal functional groups within communities, despite stable alpha diversity, reflecting compositional rather than diversity‐level effects of pesticide application.

To assess how pesticide effects may vary across cropping systems, we focused on *Olea europaea* and *Prunus dulcis* , two of the most represented crops in our dataset with balanced numbers of treated and untreated soils. Although these species are not typically associated with ectomycorrhizal symbioses, we included ectomycorrhizal taxa as a sensitive indicator group to evaluate potential alterations in soil ecological integrity. Their consistent decline under pesticide exposure suggests that ectomycorrhizal‐associated functions can provide early signals of microbiome degradation, even in non‐symbiotic systems. Ectomycorrhizal fungi declined significantly in both cropping systems (*p* = 1.5 × 10^−5^ and *p* = 7.0 × 10^−4^ for *Olea* and *Prunus*, respectively; Figure S2). In contrast, pesticide application was associated with a significant increase in plant pathogenic fungi in *Olea europaea* (*p* = 8.1 × 10^−4^), whereas no such effect was observed in *Prunus dulcis* soils (Figure S2). These results indicate that the impact of pesticides on fungal guilds may depend not only on chemical inputs but also on crop identity and associated soil environments.

We then performed differential abundance testing to identify specific microbial taxa associated with pesticide use (Figure 3e–f). Several bacterial and fungal genera were significantly enriched in pesticide‐treated soils, including *Agromyces*, *Nitrospira*, *Cystoagaricus*, *Alternaria*, and *Microdochium*—some of which include known phytopathogens. In contrast, untreated soils were enriched in genera such as *Umbelopsis*, *Hydnum*, *Scleroderma*, *Novosphingobium*, and *‘IS‐44’* (*Nitrosomonadaceae*).

**FIGURE 4:**
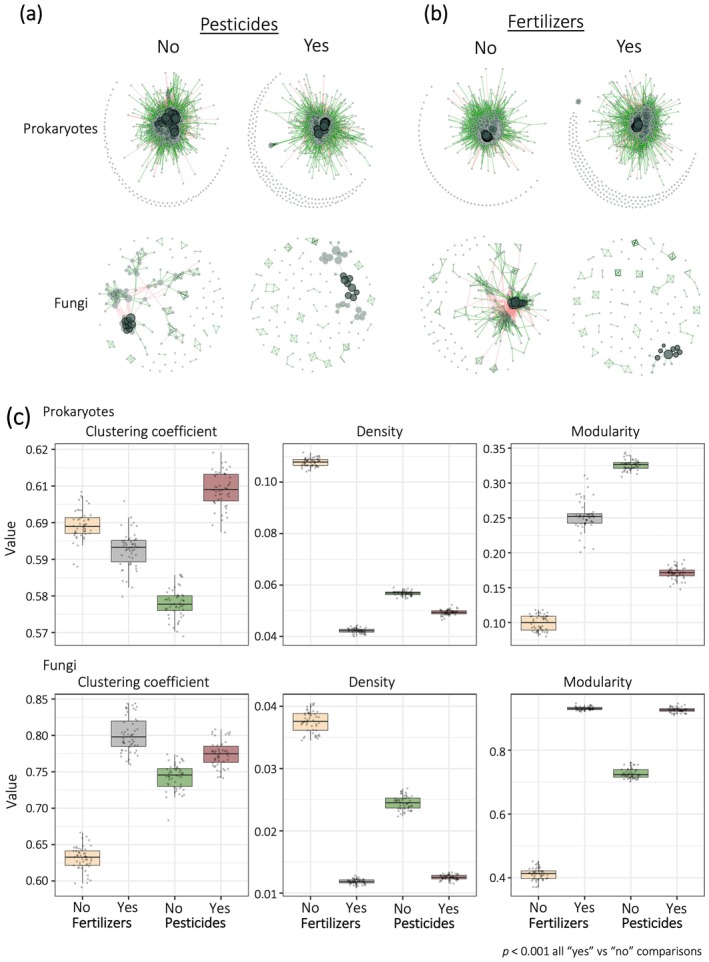
Effects of agricultural inputs on soil microbial co‐occurrence network structure. (a) Co‐occurrence networks inferred from 16S rRNA gene (top) and ITS (bottom) datasets comparing agricultural soils with versus without pesticide use. Node size represents node degree (number of connections per taxon). (b) Equivalent networks for soils with versus without fertilizer application. (c) Boxplots summarizing network‐level metrics—clustering coefficient, network density, and modularity—for the same comparisons in both datasets. All yes‐versus‐no comparisons in panel ‘c’ were highly significant (*p* < 0.001). Networks were inferred with NetCoMi using SparCC correlations (threshold = 0.3), and global metrics (clustering coefficient, network density, and modularity) were computed from 50 bootstrap replicates (see Methods for details).

We examined microbial co‐occurrence network properties (Figure 4, Table S5). In pesticide‐treated soils, network clustering coefficient increased for both 16S rRNA (*p* < 2.2 × 10^−16^) and ITS (*p* = 5.05 × 10^−12^) datasets. However, network density significantly decreased for both domains (*p* < 2.2 × 10^−16^), indicating reduced overall connectivity. Strikingly, modularity diverged between datasets: it decreased in bacterial networks but increased in fungal ones (*p* < 2.2 × 10^−16^), suggesting distinct reorganization patterns in response to pesticide disturbance.

### 3.4 Fertilization Selectively Alters Microbial Communities and Reduces Network Cohesion

We assessed the impact of fertilization on soil microbial communities. Overall alpha diversity (Shannon entropy) was not significantly affected (Figure 3g), and fertilization explained only a very small fraction of the overall community variation (*R* ^2^ = 0.004, *p* < 0.001 for both datasets; *n* = 145 unfertilized and *n* = 587 fertilized samples), suggesting minimal global effects of fertilization on microbiome composition across all soils. No clear differences were observed in the relative abundance of plant pathogenic or saprotrophic fungal guilds. However, ectomycorrhizal fungi showed a significant but moderate reduction in relative abundance in fertilized soils (Wilcoxon test, *p* = 0.004) (Figure 3h–j). Mean relative abundance decreased from 0.0521 in unfertilized soils to 0.0270 in fertilized soils (median: 0.0212 vs. 0.00568), corresponding to an approximate 48% reduction. In contrast, occurrence rates remained similar between treatments (83.4% vs. 86.7%), indicating that fertilization mainly affected abundance rather than distribution. This pattern was consistent across the two main cropping systems analyzed ( *Olea europaea* and *Prunus dulcis* ), which showed similar responses in ectomycorrhizal and pathogenic fungal guilds (Figure S2).

Differential abundance analysis revealed specific microbial responses to fertilization (Figure 3k–l). Fertilized soils were significantly enriched in several bacterial (e.g., *Thermoactinomyces*, *Ureibacillus*, *Agromyces*) and fungal (e.g., *Equiseticola, Candida*, *Itersonilia*, *Schizothecium*) genera. In contrast, unfertilized soils showed higher abundance of taxa such as *Ovatospora*, *Arachniotus*, *Knufia*, *Hymenogaster*, *Crosiella*, *Modestobacter*, and *Actinomycetospora*.

Fertilization reduced the clustering coefficient in bacterial networks (*p* = 6.49 × 10^−11^) but increased it in fungal networks, indicating contrasting effects of fertilization on local association patterns. In both domains, fertilization consistently decreased network density and increased modularity (*p* < 2.2 × 10^−16^), leading to a general fragmentation of microbial networks into more clearly defined substructures (Figure 4, Table S5). These changes may reflect a shift toward compartmentalized microbial organization under fertilization regimes, with fewer but more internally cohesive microbial clusters.

### 3.5 Contrasting Impacts of Organic and Inorganic Inputs on Soil Microbial Communities

We compared the effects of inorganic and organic fertilization on soil microbial communities. Alpha diversity was slightly higher under organic fertilization in the ITS dataset, although the difference was not significant in the 16S rRNA gene dataset (*n* = 347 organic and *n* = 215 inorganic) (Figure 3m). Notably, organic fertilizers increased the relative abundance of ectomycorrhizal and pathogenic fungi (*p* = 5.4 × 10^−7^ and *p* = 0.012, respectively), while saprotrophic fungi remained unaffected (Figure 3n–p). No significant effects were detected in the abundance of fungal pathogens or ectomycorrhizal fungi associated with *Olea europaea* or *Prunus dulcis* soils (Figure S2).

Differential abundance analysis revealed that organic fertilization enriched several bacterial taxa within *Turicibacter*, *Oceanobacillus*, *Thermoactinomycetaceae*, *Lysobacter*, and *Ureibacillus*, as well as the fungi belonging to *Pseudopithomyces*, *Setophaeosphaeria*, and *Pseudoarthrographis* (Figure 3q,r). In contrast, inorganic fertilized soils showed a higher abundance of fungal genera such as *Clitopilus*, an unclassified *Microbotryomycetes* taxon, *Occultifur*, *Apodus*, *Equiseticola*, and the bacteria *Geobacter*, *Anaeromyxobacter*, and an unclassified *Pedosphaeraceae* genus (Figure 3q,r). These patterns highlight divergent microbial recruitment under different fertilization regimes, with organic inputs promoting taxa typically associated with plant residues and nutrient cycling. In contrast, inorganic fertilizers favor a distinct subset of copiotrophic or stress‐tolerant microorganisms.

**FIGURE 5:**
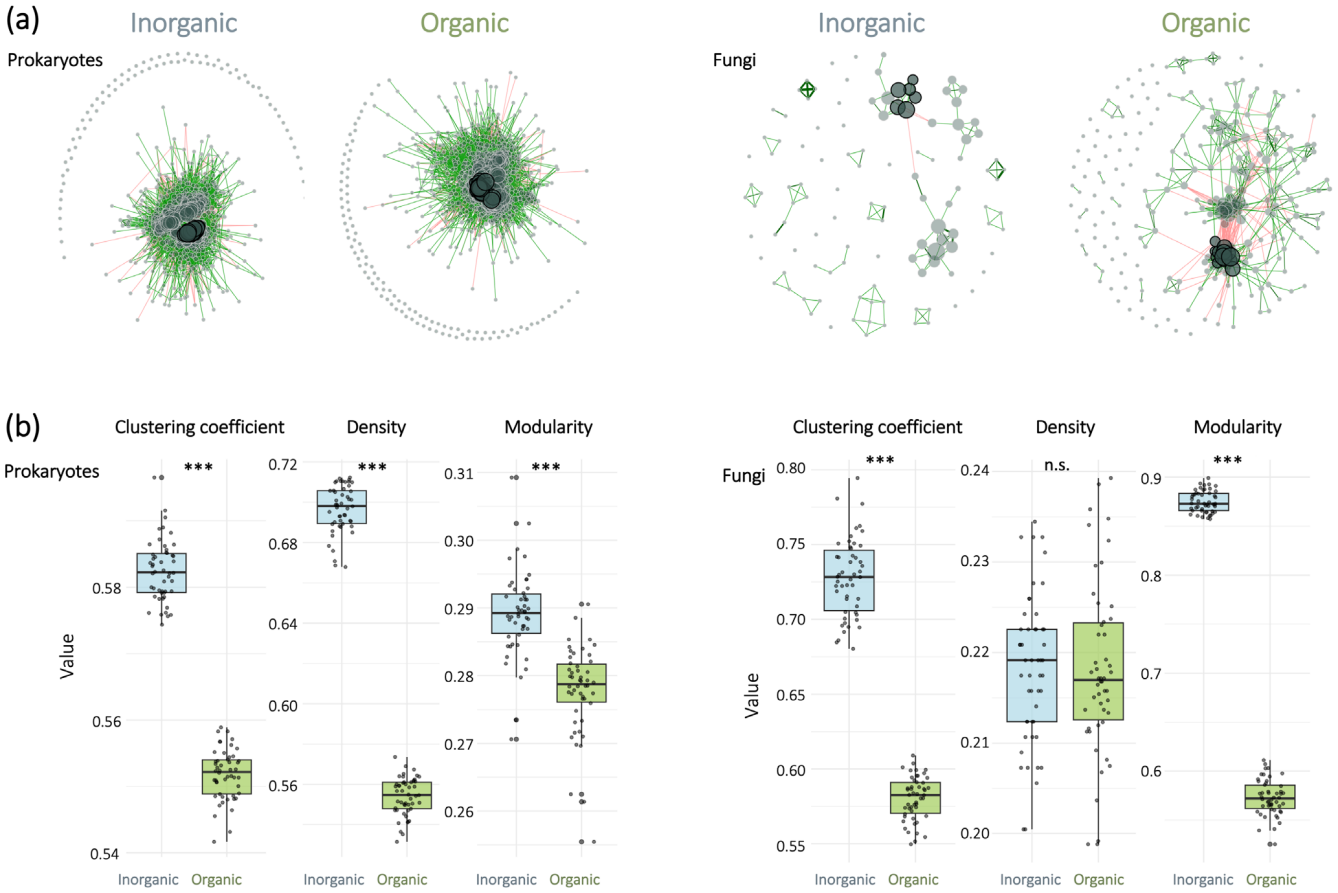
Effects of fertilizer type on soil microbial co‐occurrence network structure. (a) Co‐occurrence networks inferred from 16S rRNA gene (top) and ITS (bottom) datasets comparing soils fertilized with inorganic versus organic inputs. Node size represents node degree (number of connections per taxon). (b) Boxplots summarizing network‐level metrics—clustering coefficient, network density, and modularity—for the same comparisons in both datasets. ***p < 0.001; n.s., not significant. Network inference and metric computation followed the same NetCoMi–SparCC pipeline described in Methods.

Interestingly, microbial networks from soils treated with organic fertilizers exhibited consistently lower values of network density, clustering coefficients, and modularity in the 16S rRNA gene dataset, and reduced clustering coefficient and modularity in the ITS dataset, compared to soils fertilized with inorganic inputs (Figure 5, Table S5). This pattern indicates that organic amendments exert a pronounced desynchronizing or destructuring effect on microbial communities. This may be due to their more complex and heterogeneous composition.

## 4 Discussion

Our global analysis reveals that agricultural land use consistently drives changes in soil microbial communities, with consequences for taxonomic composition and ecological structure. Agricultural soils differed markedly from natural soils in both prokaryotic and fungal communities, showing clear shifts in dominant taxa and functional guilds. Alpha diversity was higher in agricultural soils for both domains; however, this increase was associated with the enrichment of copiotrophic, stress‐tolerant, or pathogen taxa such as *Fusarium*, *Nitrospira*, and *Agromyces*. In contrast, natural soils harbored a greater abundance of oligotrophic and symbiotic taxa, including ectomycorrhizal fungi and members of the *Burkholderia* complex. The consistent enrichment of specific prokaryotic and fungal genera and the contrasting distribution of ecological guilds suggest that deterministic processes such as those linked to nutrient availability, disturbance, and carbon inputs may play a major role in structuring community composition. At the same time, land use explains only a modest fraction of total community variance, implying that additional abiotic and biotic factors—some of which may operate in a more stochastic or context‐dependent manner—contribute to microbial turnover. These patterns support and extend previous findings at local scales (Li et al. 2022; Louisson et al. 2023; Weigel et al. 2023), and illustrate how land use acts as an ecological filter (Causevic et al. 2025; Tang et al. 2021; Li et al. 2022). In semiarid soils of southern Europe, bacterial diversity has been shown to negatively correlate with soil organic carbon—typically lower in agricultural soils—supporting the view that nutrient depletion and carbon limitation constrain prokaryotic diversity under intensive land use (Catania et al. 2022). Supporting this view, variance partitioning analyzes performed by Weigel and collaborators demonstrate that land use and spatial location together explain nearly 70% of the variation in abiotic environmental variables that act as strong predictors of microbial community structure (Weigel et al. 2023). Similar large‐scale analyzes show that both universal abiotic factors (e.g., soil pH and calcium) and ecosystem‐specific drivers shape microbial communities, with deterministic processes predominantly structuring abundant and generalist taxa, while stochastic processes have greater influence on rare and specialist taxa (Riddley et al. 2025). Previous studies have shown that both deterministic and stochastic processes can influence microbial community assembly, with deterministic processes becoming more important when communities are strongly shaped by environmental constraints (Cao et al. 2016; Dumbrell et al. 2010; Zhang et al. 2016). The observed restructuring of microbial networks and symbiotic strategies is consistent with frameworks of filtering, disturbance response, and symbiosis loss (Knight et al. 2024; Ramirez‐Villacis et al. 2025; Riddley et al. 2025).

In agricultural soils, microbial co‐occurrence networks respond differently depending on the domain and the type of agricultural input. Here we observe an increase in the clustering coefficient of bacterial communities exposed to pesticides, accompanied by a decrease in modularity, suggesting greater local interconnectivity among taxa and a reduced segregation into distinct functional or ecological modules (Faust and Raes 2012; Cardona et al. 2016; Guo et al. 2022). In contrast, fungal communities exposed to pesticides exhibit increased modularity along with increased clustering, indicating a higher degree of compartmentalization into distinct subnetworks, each possibly specialized in different functional roles related to xenobiotic degradation or stress adaptation. Similarly, fertilization leads to increased modularity and clustering coefficient in both bacterial and fungal networks, suggesting that nutrient inputs could promote the formation of more compartmentalized, functionally specialized modules with strong local cohesion. We propose that this compartmentalization may reflect changes in microbial community organization under fertilization regimes. Across treatments and domains, we observe consistent reductions in network density, reflecting a general loss of overall connectivity. In ecological terms, this decrease in density may indicate reduced functional redundancy and potentially compromised community resilience (De Vries et al. 2018; Guo et al. 2022). While some aspects of network organization become more compartmentalized, the overall fragmentation—especially the loss of connectivity—may indicate changes in the organization of soil microbiomes under continued agricultural disturbance. In agreement with these network‐level patterns, previous studies have shown that chemical fertilization can alter the abundance of individual microbial taxa through changes in soil chemistry, rather than by increasing overall microbial richness or diversity (Dincă et al. 2022). Our findings also align with previous work showing that increasing land‐use intensity can reduce the size and complexity of microbial networks and alter the balance between positive and negative associations (Romdhane et al. 2022). This reinforces the notion that agricultural disturbance restructures microbial interactions across domains and reshapes ecological networks in complex, input‐ and domain‐specific ways.

Our results reveal that fungi and bacteria exhibit contrasting patterns of diversity and community restructuring in response to both climate and agricultural management. These divergent responses may reflect fundamental differences in life‐history strategies and ecological roles between these domains, as suggested by patterns of differential sensitivity to environmental and land‐use factors (Kaisermann et al. 2015; Lan et al. 2022; Wang et al. 2022; Labouyrie et al. 2023). Recognizing this domain‐specific sensitivity may help improve our understanding of how different components of the soil microbiome respond to changing land‐use regimes and associated environmental pressures.

The results confirm recent evidence showing that conversion from conventional to organic management drives distinct shifts in soil microbial communities, although not necessarily increasing overall bacterial diversity (van Rijssel et al. 2022). Compared to inorganic fertilization, organic amendments altered fungal community composition, promoting ectomycorrhizal and pathogenic fungi, while having no significant effect on saprotrophic groups. While local studies have reported contrasting outcomes regarding the effects of organic fertilization on fungal trophic guilds and other microbial traits (e.g., Soonvald et al. 2019; Stiborova et al. 2021; Su et al. 2022), our global patterns better capture the prevailing tendencies across diverse agroecosystems. The observed reduction in microbial network density, clustering, and modularity under organic fertilization indicates changes in network topology compared with inorganic fertilized soils. Reduced modularity and clustering suggest a less compartmentalized and less tightly connected network structure (Faust and Raes 2012; Cardona et al. 2016; Guo et al. 2022), without implying direct causal relationships with environmental heterogeneity or resource availability.

The consistent taxonomic shifts associated with agricultural management may reflect legacy effects or ecological hysteresis (Ratajczak et al. 2018). Even in the absence of recent disturbances, microbial assemblages could remain shaped by historical inputs, crop types, and management regimes. These legacy effects may have implications for the reversibility of agricultural impacts and the potential for soil microbiomes to recover after transitions to more sustainable practices. For instance, among all microbial groups analyzed, ectomycorrhizal fungi consistently declined in relative abundance across pesticide and fertilization use, and organic versus inorganic amendments. These taxa were also significantly more abundant in natural soils, suggesting that they are particularly sensitive to anthropogenic disturbance. Given their key role in plant nutrient acquisition and carbon cycling, their depletion could have cascading effects on ecosystem functioning. Ectomycorrhizal fungi are known to be highly dependent on stable plant hosts, complex soil organic matter, and minimal chemical interference—conditions often disrupted in managed soils (van der Linde et al. 2018; Jörgensen et al. 2024). As such, they may serve as bioindicators of ecological integrity and early warning signals of microbiome degradation in agricultural landscapes. It should be noted that, despite their widespread use and generally conservative ecological meaning, functional trait assignments inferred from taxonomy may not fully capture the heterogeneity within microbial lineages. Substantial within‐genus and within‐species strain diversity—particularly in bacteria (Li et al. 2021; Koga et al. 2022; Saati‐Santamaría et al. 2022, 2025), but also in fungi (Naranjo‐Ortiz and Gabaldón 2019; Rai and Agarkar 2016; Sauters and Rokas 2025)—can result in distinct, or even opposing, ecological roles, such as transitions between saprotrophic, pathogenic, or mutualistic lifestyles, introducing some uncertainty into broad guild‐level interpretations.

The finding that microbial alpha diversity was higher in agricultural soils than in natural ones, for both prokaryotes and fungi, may be related to the effects of moderate, recurrent disturbances associated with cultivation. Periodic soil disturbance, fertilization, and inorganic inputs can generate spatial and temporal heterogeneity that may promote coexistence by reducing competitive exclusion. This pattern resembles predictions from the intermediate disturbance hypothesis (IDH), although the general validity of this framework is under debate and alternative diversity–disturbance relationships have been reported (Miller et al. 2011; Fox 2013; Sheil and Burslem 2013). Importantly, increased alpha diversity does not necessarily imply enhanced ecosystem function, particularly if the additional taxa are opportunistic or pathogenic rather than mutualistic or functionally redundant.

## 5 Conclusion

In summary, our global analysis demonstrates that agricultural management exerts a pervasive influence on the structure, diversity, and connectivity of soil microbial communities. These effects are expressed not only through compositional shifts but also through fundamental reorganization of microbial interactions and guild dynamics. Across biomes, we find that agricultural practices—particularly pesticide use and fertilization—reshape microbial networks in ways that may compromise long‐term resilience. The consistent shifts in microbial traits or taxa and the reorganization of microbial networks highlight the vulnerability of mutualistic interactions under intensive management. The findings consistently demonstrate a negative functional shift: agricultural inputs of all types (including organic) increase the abundance of pathogens and, crucially, cause a widespread decline in vital symbiotic fungi, such as ectomycorrhizal fungi. At the same time, elevated α‐diversity in agricultural soils and context‐dependent responses indicates that moderate disturbance and resource heterogeneity may foster microbial coexistence, albeit with potential trade‐offs for ecosystem stability. We acknowledge that, as with all large‐scale observational studies, other unmeasured environmental factors—such as soil physicochemical properties and interactions with other components of the soil biota—may also contribute to the observed patterns. Together, these findings provide a framework for understanding how soil management affects the structure of belowground biodiversity. Our results also emphasize the need to design agricultural systems that balance productivity with the conservation of soil microbiome integrity, thus supporting sustainable and resilient agroecosystems.

## Author Contributions

**Zaki Saati‐Santamaría:** methodology, conceptualization, investigation, writing – original draft, visualization, writing – review and editing, software, formal analysis, data curation, resources, supervision. **Ewa Lojkowska:** investigation, writing – review and editing. **Weronika Babinska‐Wensierska:** investigation, writing – review and editing. **David Marcos‐Vidal:** investigation, writing – review and editing. **Sergio Pérez‐Gorjón:** conceptualization, investigation, methodology, project administration, supervision, resources, data curation, writing – review and editing, validation. **Francisco Beitia:** investigation, writing – review and editing. **Amanda Lucia Alves:** investigation, writing – review and editing. **Jorge Ariel Marfetán:** investigation, writing – review and editing. **María Belén Pildain:** investigation, writing – review and editing. **Daniel Pinto‐Carrasco:** investigation, writing – review and editing. **Daniel Abel‐Schaad:** investigation, writing – review and editing. **Carolina Barroetaveña:** investigation, writing – review and editing. **Natalia Rosas‐Ramos:** investigation, writing – review and editing. **David Rodríguez de la Cruz:** investigation, writing – review and editing. **Francisca Alba‐Sánchez:** investigation, writing – review and editing. **Gonzalo M. Romano:** investigation, writing – review and editing. **Patricia Vieira Tiago:** investigation, writing – review and editing. **Alberto Acedo‐Bécares:** investigation, writing – review and editing. **Kara Barry:** investigation, writing – review and editing. **María Laura Vélez:** investigation, writing – review and editing. **Gregory Bonito:** investigation, writing – review and editing. **Guillermo Cabezas:** investigation, writing – review and editing. **Katerina Biniari:** investigation, writing – review and editing. **Philippe Delavault:** investigation, writing – review and editing. **Martina Ferraguti:** investigation, writing – review and editing. **Victoria Bueno‐González:** investigation, writing – review and editing. **Parharidis Charalambos:** investigation, writing – review and editing. **Andrés de Errasti:** investigation, writing – review and editing. **Paula García‐Fraile:** investigation, writing – review and editing. **Luis de Pedro Noriega:** investigation, writing – review and editing. **Mihalis Boutaris:** investigation, writing – review and editing. **David González del Pozo:** investigation, writing – review and editing. **Ana Laura Gallo:** investigation, writing – review and editing. **Abel Fernández‐Ruiz:** investigation, writing – review and editing. **Fernando Dianez‐Martínez:** investigation, writing – review and editing. **Mario Garrido:** investigation, writing – review and editing. **Gabriel Grilli:** investigation, writing – review and editing. **Ovidiu Copoț:** investigation, writing – review and editing. **Edmundo Danilo Guilcapi‐Pacheco:** investigation, writing – review and editing. **Danny Haelewaters:** investigation, writing – review and editing. **Alfredo Justo:** investigation, writing – review and editing. **Kentaro Hosaka:** investigation, writing – review and editing. **Andrés Hirigoyen:** investigation, writing – review and editing. **Pablo Yair Huais:** investigation, writing – review and editing. **Alina G. Greslebin:** investigation, writing – review and editing. **Marjo Keskitalo:** investigation, writing – review and editing. **Marja Jalli:** investigation, writing – review and editing. **Terry W. Henkel:** investigation, writing – review and editing. **Elí Misael Bobadilla‐Peñaló:** investigation, writing – review and editing. **Enrico Ercole:** investigation, writing – review and editing. **Antonio J. Mendoza‐Fernández:** investigation, writing – review and editing. **David Diez‐Méndez:** investigation, writing – review and editing. **Lucía Molina:** investigation, writing – review and editing. **Isabel Miralles‐Mellado:** investigation, writing – review and editing. **Aneta Lambevska:** investigation, writing – review and editing. **Corina Leconte:** investigation, writing – review and editing. **Norman Muzhinji:** investigation, writing – review and editing. **Ewald Langer:** investigation, writing – review and editing. **Francisco J. Oficialdegui:** investigation, writing – review and editing. **Ansa Palojärvi:** investigation, writing – review and editing. **Raúl Ortega Pérez:** writing – review and editing, investigation. **Diego Nieto‐Lugilde:** investigation, writing – review and editing. **Tommaso La Mantia:** investigation, writing – review and editing. **André‐Ledoux Njouonkou:** writing – review and editing. **Minh N. Nguyen:** investigation, writing – review and editing. **Marcos Paradelo‐Pérez:** investigation, writing – review and editing. **Aída M. Vasco‐Palacios:** investigation, writing – review and editing. **Anna Maria Persiani:** investigation, writing – review and editing. **Sarah Norvell:** investigation, writing – review and editing. **Lucie Poulin:** investigation, writing – review and editing. **Julio Peñas de Giles:** investigation, writing – review and editing. **Paola Quatrini:** investigation, writing – review and editing. **Isabel Salcedo‐Larralde:** investigation, writing – review and editing. **Zunilda Pavone:** investigation, writing – review and editing. **Alan R. Wood:** investigation, writing – review and editing. **Mylonas Vasilis:** investigation, writing – review and editing. **Nourou S. Yorou:** investigation, writing – review and editing. **Ricardo Valenzuela:** investigation, writing – review and editing. **Jean‐Bernard Pouvreau:** investigation, writing – review and editing. **Alberto Nieto‐Palenzuela:** investigation, writing – review and editing. **Cathy Sharp:** investigation, writing – review and editing. **Francisca Ruano:** investigation, writing – review and editing. **Esteban Salmerón‐Sánchez:** investigation, writing – review and editing. **Sergey Volobuev:** investigation, writing – review and editing. **Katerina Sam:** investigation, writing – review and editing. **Pirjo Yli‐Hemminki:** investigation, writing – review and editing. **Ivan V. Zmitrovich:** investigation, writing – review and editing. **Alfredo Vizzini:** investigation, writing – review and editing. **Javier Bobo‐Pinilla:** conceptualization, investigation, methodology, validation, writing – review and editing, project administration, resources, supervision.

## Conflicts of Interest

The authors declare no conflicts of interest.

## Supporting information


**Figure S1:** Relationships between Shannon entropy and all tested bioclimatic variables for 16S rRNA gene and ITS datasets, modeled using generalized additive models (GAMs). These analyzes extend the patterns shown in Figure 1c for annual mean temperature and mean temperature of the driest quarter to the full set of bioclimatic predictors.

**Figure S2:** Relative abundance of fungal functional guilds under pesticide and fertilization regimes in *Olea europaea* and *Prunus dulcis* soils. Relative abundance of plant‐pathogenic and ectomycorrhizal fungi in soils subjected to (a,b) pesticide application, (c,d) fertilizer use, and (e,f) inorganic versus organic fertilization. Statistical significance was determined using two‐sided Wilcoxon tests with Benjamini–Hochberg correction for multiple comparisons (*p* < 0.05, *p* < 0.01, *p* < 0.001).

**Table S1:** Metadata and environmental variables for soil samples included in this study. The table provides geographic, ecological, and management information for each soil sample, including location, biome classification, land use, crop species, and records of pesticide and fertilizer application. Bioclimatic variables (bio1–bio19) correspond to the WorldClim dataset and represent temperature and precipitation‐related parameters used in diversity and community analyzes.

**Table S2:** Summary of 16S rRNA amplicon sequencing data processing, showing read counts after filtering, denoising, and chimera removal, and the proportion of reads retained at each step.

**Table S3:** Summary of ITS amplicon sequencing data processing, showing read counts after filtering, denoising, and chimera removal, and the proportion of reads retained at each step.

**Table S4:** Climatic variables ranked according to the proportion of variance explained (*R* ^2^) in prokaryotic (16S) and fungal (ITS) Shannon entropy. Climate variables correspond to WorldClim bioclimatic predictors (BIO1–BIO19), and *R* ^2^ values were obtained from generalized additive models (GAMs) fitted independently for each variable and marker.

**Table S5:** Network properties of fungal (ITS) and prokaryotic (16S rRNA) microbial co‐occurrence networks under different agricultural management practices (pesticide use, fertilizer use, and fertilizer type).

## Data Availability

Raw sequence data obtained through amplicon sequencing are available at the NCBI Sequence Read Archive (SRA) database under the Bioproject PRJNA1364335. Processed data supporting this study are available in a Zenodo repository (DOI: https://doi.org/10.5281/zenodo.18502016), including sample metadata with environmental and climatic variables, and genus‐level relative abundance tables for bacterial (16S rRNA) and fungal (ITS) communities.
