# Supplementary figures and images for: Soil Microbial Diversity and Network Organization Respond to Land Use and Agricultural Inputs Worldwide
Source: Glob Chang Biol. 2026 Jul 4;32(7):e70984. doi: 10.1111/gcb.70984 (PMC13332421; doi:10.1111/gcb.70984)

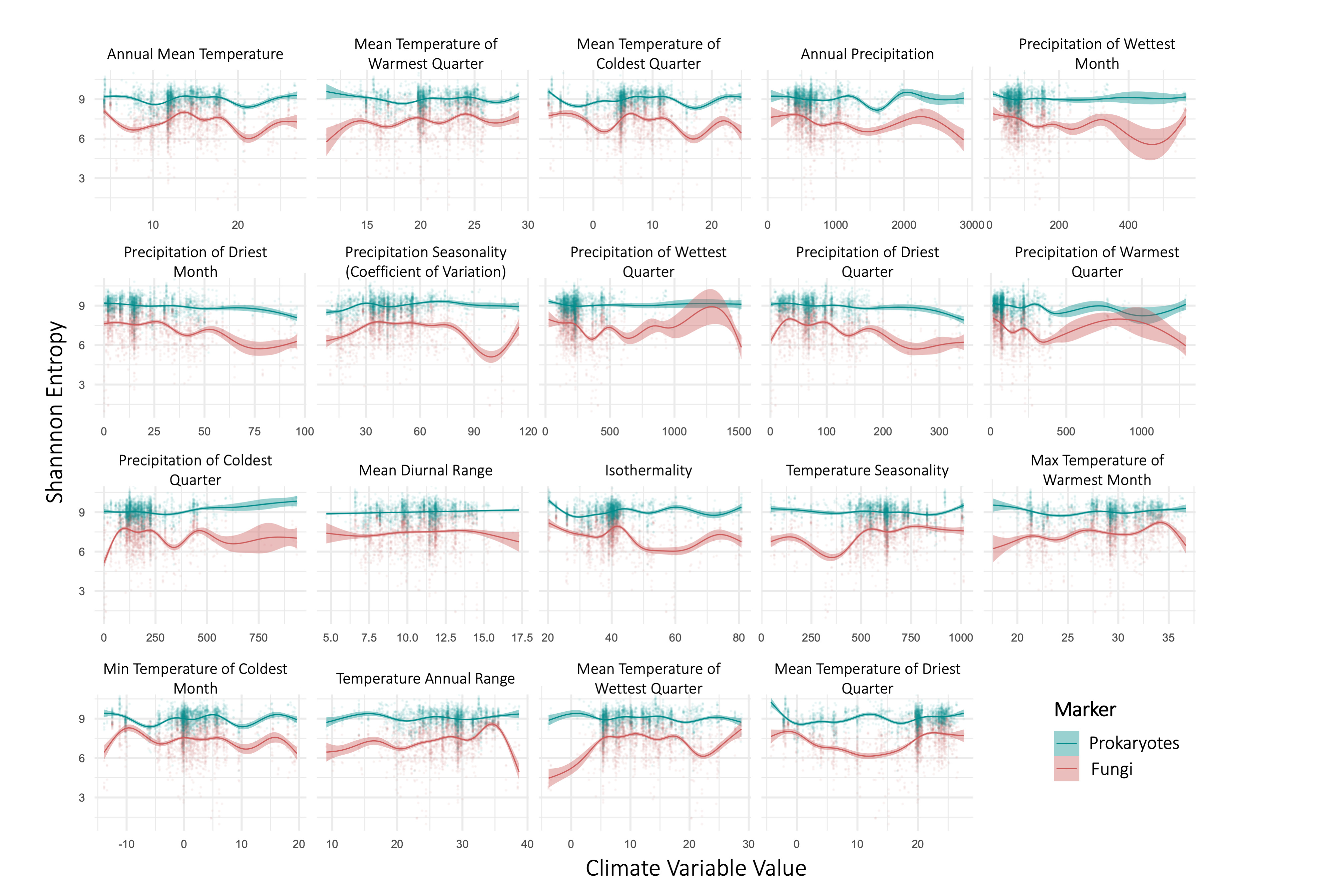

Supplement: Supplementary file 1 — Figure S1: Relationships between Shannon entropy and all tested bioclimatic variables for 16S rRNA gene and ITS datasets, modeled using generalized additive models (GAMs). These analyzes extend the patterns shown in Figure 1c for annual mean temperature and mean temperature of the driest quarter to the full set of bioclimatic predictors. [file GCB-32-e70984-s003.tiff]

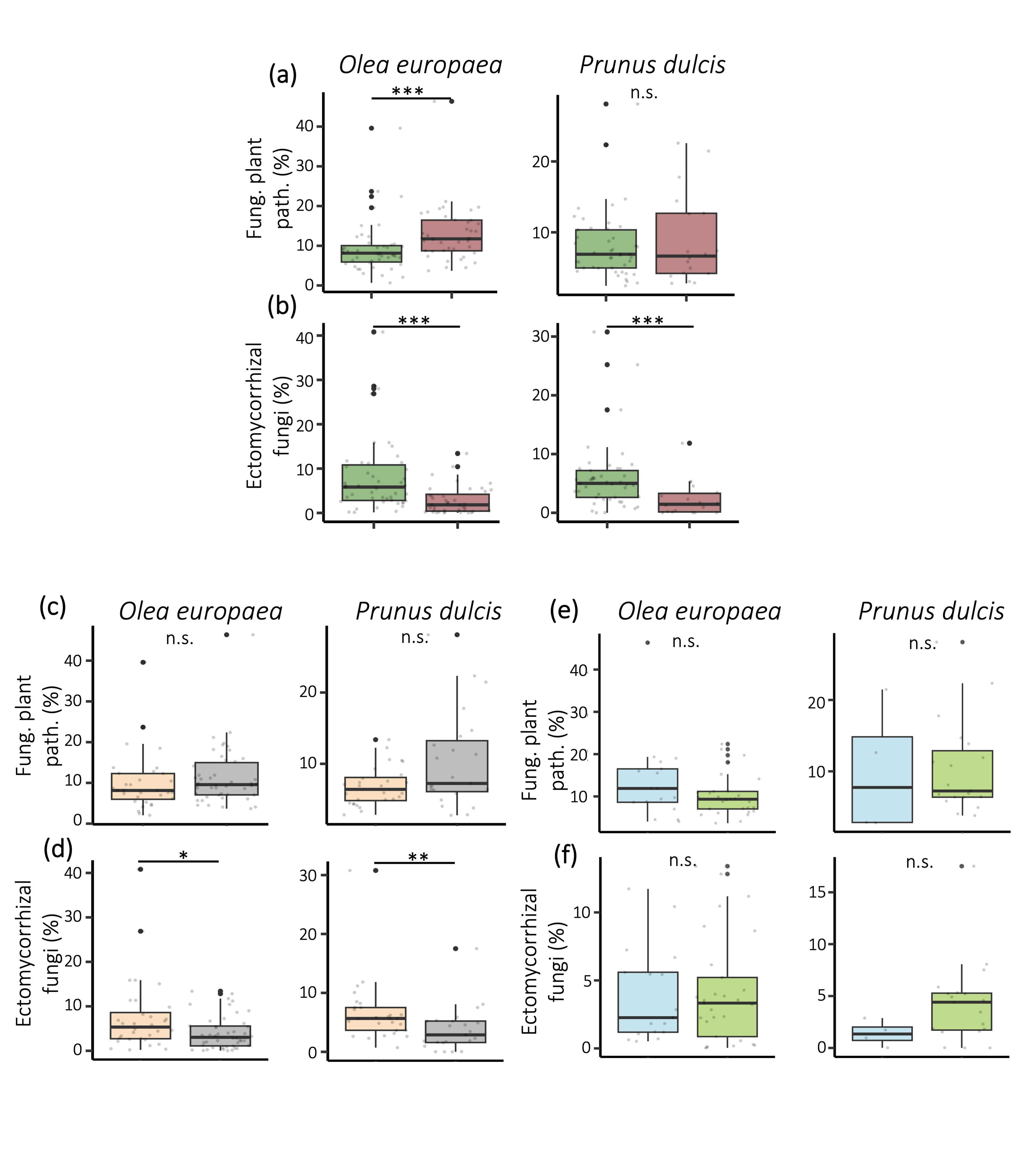

Supplement: Supplementary file 2 — Figure S2: Relative abundance of fungal functional guilds under pesticide and fertilization regimes in Olea europaea and Prunus dulcis soils. Relative abundance of plant‐pathogenic and ectomycorrhizal fungi in soils subjected to (a,b) pesticide application, (c,d) fertilizer use, and (e,f) inorganic versus organic fertilization. Statistical significance was determined using two‐sided Wilcoxon tests with Benjamini–Hochberg correction for multiple comparisons (p < 0.05, p < 0.01, p < 0.001). [file GCB-32-e70984-s002.tiff]
